# The role of red cell distribution width in the locoregional recurrence of laryngeal cancer^☆^

**DOI:** 10.1016/j.bjorl.2018.03.004

**Published:** 2018-04-13

**Authors:** Gülpembe Bozkurt, Arzu Yasemin Korkut, Pınar Soytaş, Senem Kurt Dizdar, Zeynep Nur Erol

**Affiliations:** a Acibadem University, School of Medicine, Department of Otolaryngology, Head and Neck Surgery, Istanbul, Turkey; b Sisli Hamidiye Etfal Education and Research Hospital, Department of Otolaryngology, Istanbul, Turkey; c Hopa State Hospital, Department of Otolaryngology, Artvin, Turkey

**Keywords:** Red cell distribution width, Larynx cancer, Disease free survival, Amplitude de distribuição de eritrócitos, Câncer de laringe, Sobrevida livre de doença

## Abstract

**Introduction:**

Although the red cell distribution width has been reported as a reliable predictor of prognosis in several types of cancer, to our knowledge few reports have focused on the prognostic value of red cell distribution width in laryngeal carcinoma.

**Objective:**

We aimed to explore whether the pretreatment red cell distribution width predicted recurrence in laryngeal cancer patients is a simple, reproducible, and inexpensive prognostic biomarker.

**Methods:**

All laryngeal cancer patients who underwent curative surgery (*n* = 132) over a 7 year study period were evaluated. Data on demographics, primary tumor site, T-stage, N-stage, histological features (differentiation; the presence of perineural/perivascular invasion), treatment group (total laryngectomy or partial laryngectomy) or adjuvant therapy (chemotherapy/radiotherapy); laboratory parameters (complete blood count, including the pre-operative red cell distribution width), and disease-free survival rates were retrospectively reviewed. All cases were divided into three groups by the red cell distribution width tertile [<13% (25th percentile) (*n* = 31), 13–14.4% (50th percentile) (*n* = 72), and >14.4% (75th percentile) (*n* = 29)].

**Results:**

High-red cell distribution width group included more patients of advanced age, and more of those with recurrent and metastatic tumors (*p* = 0.005, 0.048, and 0.043, respectively). Individuals with red cell distribution width >14.4% (75th percentile) had lower disease free survival rates than did those with red cell distribution width <13% (25th percentile) (*p* = 0.014). Patients with red cell distribution width >14.4% at diagnosis were at a higher risk of locoregional recurrence (hazard ratio = 5.818, 95% confidence interval (95% CI) 1.25–26.97; *p* = 0.024) than patients with a normal red cell distribution width (<13%).

**Conclusion:**

We found that the pretreatment red cell distribution width was independently prognostic of disease free survival rate in patients with laryngeal cancer and may serve as a new, accurate, and reproducible means of identifying early-stage laryngeal cancer patients with poorer prognoses.

## Resumo

**Introdução:**

Embora a amplitude de distribuição de eritrócitos tenha sido relatada como um preditor confiável de prognóstico em vários tipos de câncer, que seja de nosso conhecimento, poucos estudos se concentraram no valor prognóstico dessa medida no carcinoma laríngeo.

**Objetivo:**

Avaliar se a amplitude de distribuição de eritrócitos pré-tratamento prevê a recorrência em pacientes com câncer de laringe como um biomarcador prognóstico simples, reprodutível e não dispendioso.

**Método:**

Foram avaliados todos os pacientes com câncer de laringe submetidos à cirurgia curativa (n = 132) durante sete anos. Dados demográficos, local do tumor primário, estágio T, estágio N, características histológicas (diferenciação; presença de invasão perineural/perivascular), grupo de tratamento (laringectomia total ou laringectomia parcial) ou terapia adjuvante (quimioterapia/radioterapia); parâmetros laboratoriais (hemograma, inclusive a amplitude de distribuição de eritrócitos pré-operatório) e a sobrevida livre de doença foram revisados retrospectivamente. Todos os casos foram divididos em três grupos pelo tercil da amplitude de distribuição de eritrócitos [< 13% (percentil 25) (n = 31), 13% – 14,4% (percentil 50) (n = 72) e > 14,4% (percentil 75) (n = 29)].

**Resultados:**

O grupo com amplitude de distribuição de eritrócitos elevado incluiu um número maior de pacientes com idade avançada e mais pacientes com tumores recorrentes e metastáticos (*p* = 0,005, 0,048 e 0,043, respectivamente). Os indivíduos com a amplitude de distribuição de eritrócitos >14,4% (percentil 75) apresentaram taxas de sobrevida livre de doença menores do que aqueles com a amplitude de distribuição de eritrócitos <13% (percentil 25) (*p* = 0,014). Pacientes com a amplitude de distribuição de eritrócitos >14,4% no diagnóstico apresentaram maior risco de recorrência locorregional [*Hazard Ratio* = 5,818, intervalo de confiança de 95% (IC 95%) 1,25–26,97; *p* = 0,024] do que pacientes com a amplitude de distribuição de eritrócitos normal (< 13%).

**Conclusão:**

Verificamos que a amplitude de distribuição de eritrócitos pré-tratamento foi um fator prognóstico independente de sobrevida livre de doença em pacientes com câncer de laringe e pode servir como um novo parâmetro, preciso e reprodutível, para identificar pacientes com câncer de laringe em estágio inicial com piores prognósticos.

## Introduction

In recent years, systemic hematological markers have become increasingly recognized as prognostic of the outcomes of malignancies. The red cell distribution width (RDW), routinely measured during a complete blood cell count (CBC), reflects the extent of erythrocyte size heterogeneity (i.e., the level of anisocytosis). RDW is a marker of the systemic inflammatory response, and many studies have explored the relationship between RDW and cardiovascular disease and inflammatory conditions.^1^, ^2^, ^3^, ^4^, ^5^, ^6^ Recently, RDW was shown to be of prognostic utility in patients with different malignancies.^7^, ^8^, ^9^ An earlier study indicated that the RDW might predict the survival of laryngeal cancer patients.^10^

Laryngeal Squamous Cell Cancer (LSCC) is one of the most common malignancies worldwide, and the identification of prognostic factors may improve patient outcomes. Known prognostic factors include T-stage, an extracapsular extension, neck involvement, and surgical treatment outcomes.^11^, ^12^ These factors are disease stage-associated; little is known about non-disease-related prognostic factors. Although certain molecular signatures have been used to stratify survival in different cohorts of LSCC patients,^13^, ^14^, ^15^ no simple, reproducible, and inexpensive prognostic biomarker for use in clinical settings is yet available. A CBC is a simple, reproducible, and inexpensive test. Thus, our purpose was to explore whether the pretreatment RDW predicted recurrence in laryngeal cancer patients.

## Materials and methods

After obtaining the approval of local Ethics Committee (No. 812-13/06/2017), we retrospectively reviewed data on 132 consecutive patients who underwent surgery to treat LSCC between 2002 and 2009 in our unit. We recorded demographic data and information on staging, pre-operative workup, treatments, and outcomes (as recorded in case notes, surgical logbooks, and our electronic patient and pathology databases). Unfortunately some pathology reports were either unclear or missing. Unclear data were omitted from analysis.

Patients with histopathologically confirmed laryngeal cancer and those who underwent curative resection (partial or total laryngectomy with neck dissection) were included. The exclusion criteria were as follows: (1) non-surgical treatment (chemotherapy/radiotherapy); (2) recurrent laryngeal cancer or another histological type of laryngeal cancer other than squamous cell carcinoma (e.g., stromal tumors and neuroendocrine tumors); (3) prescription of neoadjuvant therapy; (4) pathological downstaging of tumors to Stage I or II from Stage III or IV; (5) a cohesive front or positive excision margin; (6) any in situ malignancy, and (7) an unclear pathology report. All patients were staged using the AJCC/UICC-TNM system for laryngeal cancer (7th edition).^16^ The variables used to stratify survival included age, primary tumor site, T-stage, N-stage, histological features (differentiation; the presence of perineural/perivascular invasion), treatment group therapeutic (Total Laryngectomy –TL or Partial Laryngectomy – PL) or Adjuvant Therapy (Chemotherapy – CT/Radiotherapy – RT); laboratory parameters (CBC, including the pre-operative RDW), and disease-free survival (DFS). All patients were divided into two groups in terms of disease stage: early (T-stages I and II) (*n* = 73) and advanced (T-stages III and IV) (*n* = 59). Follow-up featured clinical examinations every 3–6 months for the first 2 years and annual examinations thereafter. This ensured a follow-up duration of ≥5 years. DFS was the duration from the date of diagnosis to the date of locoregional recurrence or the last follow-up. Only 10 patients died during follow-up (4 from cancer); overall survival could thus not be calculated. Distant Recurrence (DR) was diagnosed either clinically (including via ultrasound-guided fine needle aspiration) or via imaging (Computed Tomography – CT or positron emission CT). All recurrences were biopsy-proven except in patients in whom either comorbid disease or disease progression rendered invasive biopsies contraindicated.

### Statistics

All statistical analyses were performed with the aid of SPSS version 15.0 for Windows. Descriptive statistics are expressed as means ± standard deviations (SDs) and medians (continuous variables), or as frequencies with percentages (categorical variables). All cases were divided into three groups by the RDW tertile [<13% (25th percentile), 13–14.4% (50th percentile), and >14.4% (75th percentile)]. Clinically relevant differences were defined using the Chi-squared test or via Monte Carlo simulation, as appropriate. Continuous and categorical parameters were compared using the independent samples *t*-test or the Mann–Whitney *U* test and chi-squared test, respectively. Survival was assessed with the aid of the log-rank Kaplan–Meier method. Deaths from causes other than cancer were censored at the dates of death prior to DSS analysis. Variables that were potentially prognostic on univariate analyses were subjected to multivariate analyses using a Cox's proportional hazards regression model. A two-sided *p*-value <0.05 was considered to reflect statistical significance.

## Results

We evaluated 132 males of mean age 59.7 ± 9.4 years (range 33–83 years). The mean RDW (%) of the entire cohort was 13.7 ± 1.2 (25th–75th percentile: 13–14.4). In total, 31 patients had RDWs <13% (25th percentile), 72 RDWs 13–14.4% (50th percentile), and 29 RDWs >14.4% (75th percentile). Twenty-four patients (18.2%) developed locoregional recurrences during follow-up. During a median follow-up period of 78.6 ± 22.0 months (range 2–96 months), the overall mortality was 7.5% (*n* = 10). Most deaths were attributable to factors other than index malignancies (thus, treatment-related complications or unrelated/unknown causes). Four patients died of their diseases per se. Thirteen (9.8%) patients developed distant metastases (Table 1).

**Table 1:** Demographic data on and clinical characteristics of the patients.

Patient characteristic	Value
*Age (years), mean* *±* *SD (min–max)*	59.7 ± 9.4 (33–83)
*RDW, mean* *±* *SD (min–max)*	13.7 ± 1.2 (10.5–18.1)
*25th percentile (13)*	31 (23.5)
*50th percentile (13.6)*	72 (54.5)
*75th percentile (14.4)*	29 (22.0)
*Stage, n (%)*	
T1	41 (31.1)
T2	32 (24.2)
T3	50 (37.9)
T4	9 (6.8)

*Anatomical site, n (%)*	
Glottis	57 (43.2)
Supraglottis	49 (37.1)
Transglottis	26 (19.7)

*LAP, n (%)*	
N1	8 (6.1)
N2a	1 (0.8)
N2b	9 (6.8)
N2c	7 (5.3)
N0	107 (81.1)

*Differentiation, n (%)*	
Poor	24 (18.1)
Mild	84 (63.6)
Extensive	24 (18.2)

*Perivascular invasion, n (%)*	34 (25.8)
*Perineural invasion, n (%)*	19 (14.4)
*Distant metastasis, n (%)*	13 (9.8)
*Time to metastasis (months); mean* *±* *SD (min–max)*	35.5 ± 18.7 (6–72)
*Locoregional relapse, n (%)*	24 (18.2)
*Relapse interval (months); mean* *±* *SD (min–max)*	25.7 ± 19.2 (6–72)
*Adjuvant treatment, n (%)*	
CRT	4 (3.0)
RT	46 (34.8)
No	82 (62.1)

*Operation, n (%)*	
TL	65 (49.2)
SGL	24 (18.2)
Cordectomy	16 (12.1)
FLL	21 (15.9)
VHL	1 (0.8)
SCL	4 (3.03)
NTL	1 (0.8)

*Follow-up time (months); mean* *±* *SD (min–max)*	78.6 ± 22.0 (2–96)
*Mortality, n (%)*	
Treatment-related factors	6 (4.54)
Laryngeal cancer	4 (3.03)

*Postoperative complications, n (%)*	23 (17.4)

SD, standard deviation; LAP, lymphadenopathy; TL, total laryngectomy, SGL, supraglottic laryngectomy; FLL, frontolateral laryngectomy; VHL, vertical hemilaryngectomy; SCL, supracricoid laryngectomy; NTL, near-total laryngectomy; CRT, chemoradiotherapy; RT, radiotherapy.

The characteristics of patients stratified by the pretreatment RDW are listed in Table 2. We found no significant correlation between RDW and T-stage, tumor site, nodal involvement, the extent of histological differentiation, the level of perivascular or peritumoral invasion, surgical choice, or adjuvant chemoradiotherapy status (yes or no). However, the high-RDW group included more patients of advanced age, and more of those with recurrent and metastatic tumors (*p* = 0.005, 0.048, and 0.043, respectively). On subgroup analysis, patients with RDWs >14.4% (75th percentile) were more likely to develop locoregional recurrences than those with RDWs <13% (25th percentile) (*p* = 0.014). However, the difference between patients with RDWs >14.4% (75th percentile) and RDWs 13–14.4% (50th percentile) was not significant (*p* = 0.153). On the other hand, the high-RDW group (75th percentile) included more elderly patients than the 50th percentile group (*p* = 0.004). Distant metastasis was more frequent in the 75th percentile RDW group than the 50th percentile RDW group (17.2 vs. 4.2%; *p* = 0.042) (Table 2).

**Table 2:** Clinicopathological characteristics of laryngeal cancer patients by RDW tertile.

	RDW			*p*-Value
	<13	13–14.4	>14.4	
	Mean ± SD	Mean ± SD	Mean ± SD	
Age (years)	60.5 ± 8.6	57.5 ± 9.3	64.0 ± 9.0	**0.05**

	RDW						*p*-Value
	<13		13–14.4		>14.4		
	*n*	%	*n*	%	*n*	%	
*Stage*							
T1	8	25.8	26	36.1	7	24.1	0.304
T2	8	258	20	27.8	4	13.8	
T3	12	38.7	23	31.9	15	51.7	
T4	3	9.7	3	4.2	3	10.3	

*Stage*							
Early	16	51.6	46	63.9	11	37.9	0.053
Advanced	15	48.4	26	36.1	18	62.1	

*Anatomical site*							
Glottis	12	38.7	31	43.1	14	48.3	0.122
Supraglottis	9	29.0	32	44.4	8	27.6	
Transglottis	10	32.3	9	12.5	7	24.1	

*LAP*	4	12.9	14	19.4	7	24.1	0.533
*Differentiation*							
Poor	7	22.6	11	15.3	5	17.2	0.852
Medium	19	61.3	45	62.5	20	69.0	
Well	5	16.1	16	22.2	4	13.8	

*Perivascular invasion*	6	19.4	18	25.0	10	34.5	0.398
*Perineural invasion*	3	9.7	10	13.9	6	20.7	0.502
*Locoregional relapse*	2	6.5	13	18.1	9	31.0	**0.048**
*Distant metastasis*	5	16.1	3	4.2	5	17.2	**0.043**
*Adjuvant treatment*							
No	17	54.8	47	65.3	18	62.1	0.462
CRT	1	3.2	1	1.4	2	6.9	
RT	13	41.9	24	33.3	9	31.0	

*Operation*							
TL	18	58.1	29	40.3	18	62.1	0.359
SGL	4	12.9	17	23.6	3	10.3	
SCL	1	3.2	2	2.8	1	3.4	
Cordectomy	4	12.9	10	13.9	2	6.9	

*FLL*	3	9.7	13	18.1	5	17.2	
*VHL*	1	3.2	0	0.0	0	0.0	
*NTL*	0	0.0	1	1.4	0	0.0	

Subgroup analysis		Age	Locoregional relapse	Distant metastasis
RDW		*p*-Value	*p*-Value	*p*-Value
<13	13–14.4	0.284	0.221	0.051
	>14.4	0.285	**0.014**	1.000
13–14.4	>14.4	**0.004**	0.153	**0.042**

RDW, red cell distribution width; SD, standard deviation; LAP, lymphadenopathy; TL, total laryngectomy; SGL, supraglottic laryngectomy; FLL, frontolateral laryngectomy; VHL, vertical hemilaryngectomy; SCL, supracricoid laryngectomy; NTL, near-total laryngectomy.

**Figure 1:**
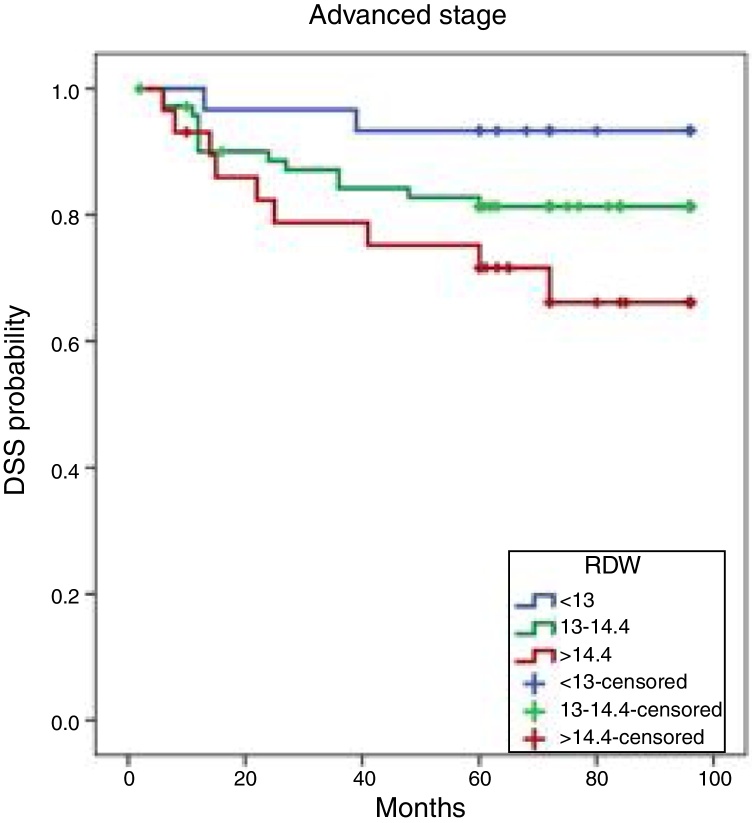
Kaplan–Meier cumulative DFS curves by RDW tertile. DFS, disease-free survival; RDW, red cell distribution width.

The 8 year DFS rates were 93.3%, 81.3%, and 66.1% among patients in the 25th, 50th, and 75th percentiles, respectively. The DFS differences among the three groups were statistically significant (*p* = 0.049). Individuals with RDWs >14.4% (75th percentile) had lower DFS rates than did those with RDWs <13% (25th percentile) (*p* = 0.014) (Fig. 1).

The RDW exhibited independent prognostic significance. The RDW at diagnosis in patients with laryngeal cancer was an independent predictor of locoregional recurrence as revealed by a multivariable analysis (Table 3). Patients with RDWs >14.4% at diagnosis were at a higher risk of locoregional recurrence (hazard ratio – HR = 5.818; 95% confidence interval – 95% CI: 1.25–26.97; *p* = 0.024) than patients with a normal RDW <13%). PL was also an independent predictor of DFS (HR = 0.233; 95% CI: 0.085–0.637; *p* = 0.005).

**Table 3:** Multivariate analysis of factors prognostic of laryngeal cancer progression as revealed by regression modeling.

	*p*-Value	HR	95% CI
**Age**	0.730	1.009	0.959–1.062
**Anatomical site (glottis)**	0.947		
*Supraglottis*	0.765	1.195	0.372–3.838
*Transglottis*	0.830	1.234	0.180–8.476

**LAP**	0.586	1.493	0.353–6.309
*Perivascular invasion*	0.300	1.828	0.584–5.725
*Perineural invasion*	0.753	0.793	0.187–3.358

**Adjuvant treatment (no)**	0.560		
*CRT*	0.982	1.029	0.085–12.427
*RT*	0.314	0.472	0.110–2.032

**Operation (PL)**	0.202	0.337	0.063–1.796
**RDW**			
*(<13)*	0.081		
*13–14.4*	0.273	2.330	0.513–10.581
*>14*	0.041	5.281	1.073–25.988

**Stages 3–4 (advanced)**	0.595	0.664	0.147–3.000
**Backward**			
*RDW*			
(<13%)	0.028		
13–14.4%	0.275	2.301	0.516–10.262
>14.4%	**0.024**	5.818	1.255–26.970

**Operation (PL)**	0.005	0.233	0.085–0.637

RDW, red cell distribution width; HR, hazard ratio; LAP, lymphadenopathy; PL, partial laryngectomy; CRT, chemotherapy; RT, radiotherapy; CI, confidence interval.

**Figure 2:**
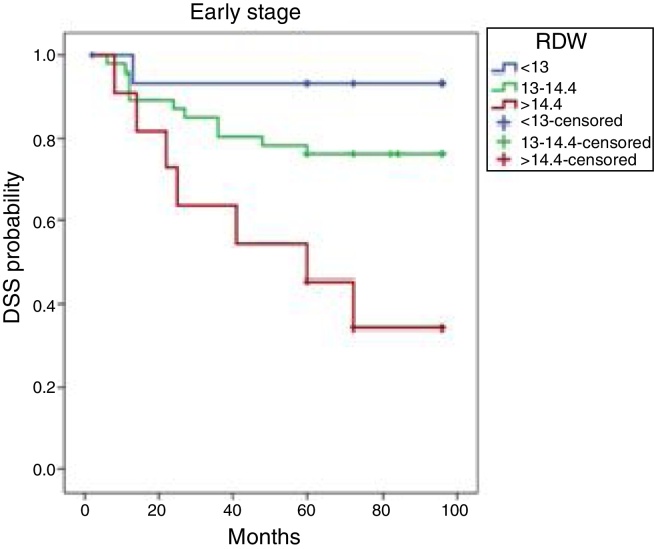
Kaplan–Meier DFS curves by RDW tertile in patients with early-stage disease. DFS, disease-free survival; RDW, red cell distribution width.

For clarity, the patient characteristics and treatment results for those treated with a curative intent are presented separately from those with T1–2 and T3–4 stage tumors. The Kaplan–Meier cumulative DFS curves based on the RDWs of early-stage patients are shown in Fig. 2. The 8 year DFS rates were 93.3, 76.1, and 34.1 in the 25th, 50th, and 75th percentile tertiles, respectively. The DFS rates of the three groups differed significantly (*p* = 0.004). At a median follow-up time of 78.6 (2–96) months, patients with RDWs <13% (25th percentile) exhibited better DFS than those with RDWs >14.4% (75th percentile) (*p* = 0.003).

**Figure 3:**
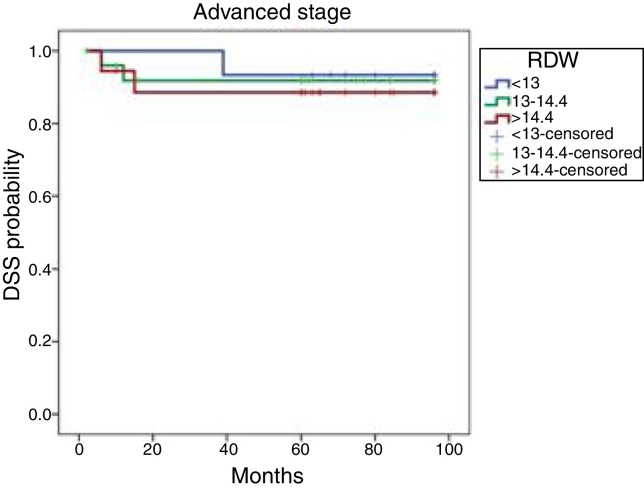
Kaplan–Meier DFS curves by RDW tertile in patients with advanced disease. DFS, disease-free survival; RDW, red cell distribution width.

The Kaplan–Meier cumulative DFS rates based on the RDWs of advanced-stage patients are shown in Fig. 3. The 8 year DFSs were 93.3%, 91.8%, and 88.5% in the 25th, 50th, and 75th percentile tertiles, respectively. These DFS rates did not differ significantly (*p* = 0.875).

## Discussion

It is widely believed that peritumoral inflammation plays crucial roles in cancer initiation and progression. Recent trials have found that easily measurable, pretreatment inflammatory markers can be used to predict Head and Neck Squamous Cell Carcinoma (HNSCC) survival and recurrence independent of the TNM stage.^17^, ^18^, ^19^ Also, elevated inflammatory markers, including the Neutrophil-to-Lymphocyte (N/L) and the platelet-to-lymphocyte ratios, were associated with poorer survival of laryngeal cancer patients.^10^, ^20^, ^21^ Inflammation inhibits red blood cell maturation by disturbing the red cell membrane, thus elevating the RDW.^22^ Many authors seek a better understanding of this relationship given the prognostic and therapeutic possibilities. RDW is a simple marker of the systemic inflammatory response and has recently been reported to negatively influence the clinical outcomes of various cancers.^23^, ^24^ However, few works have focused on HNSCC.

In this report, we present our major findings: 1) Patients with higher RDWs (>14.4%) were more likely to experience distant metastasis during treatment (*p* = 0.043) and 2) DFS was negatively correlated with the preoperative RDW. Moreover, patients with RDWs <13% (25th percentile) experienced better DFS than did patients with RDWs (>14.4%), which may thus be an appropriate threshold for the prediction of recurrence; such patients require special attention. However, Kara et al.^10^ found no difference in the RDWs of patients with local or regional recurrence, and they reported that a higher RDW increased mortality 4.6 fold and was independently prognostic of mortality in patients with laryngeal cancer. In the present study, only four of our patients died from laryngeal cancer; thus, we were unable to compare mortality rates. Chronic complications associated with prior treatment can cause mortality; many “censored” mortalities may in fact be attributable to treatment-related events. In 1990, Howell-Burke et al.^25^ made a similar observation when studying 114 patients with T2 glottic cancers, of whom only 7 died of disease. Ward et al.^26^ found that the treatment of medical comorbidities was a fundamental feature of survivorship. Our findings suggest that patient outcomes are likely explained by a combination of complex, interrelated patient-specific factors, rather than effects of the primary tumors per se. Although we found no significant Overall Survival (OS) differences, we cannot exclude the possibility that a larger series would have yielded more representative data associated with a statistically significant difference in OS rates.

Previous literature is in corroboration with our findings that the high-RDW group included more patients of advanced age.^27^, ^28^ Increased concentration of inflammatory biomarkers together with folate and vitamin B deficiencies are a part of the normal aging process,^29^ so increased RDW in the elderly should be expected. RDW also reflects anisocytosis in patients with various diseases, including cardiovascular disease, venous thromboembolism, cancer, diabetes, community-acquired pneumonia, chronic obstructive pulmonary disease, and liver and kidney failure.^30^ An increased RDW may be a feature of several human cancers. Yazici et al.^31^ found that the RDW was a potential prognostic biomarker in patients with gastric cancer. Riedl et al.^32^ reported that a high RDW independently predicted poor OS in patients with various cancers, including brain, breast, colon, kidney, or lung cancer; lymphoma and multiple myeloma; and pancreas, prostate, or stomach cancer. The RDWs of patients with colon cancer were significantly higher than those of patients with colonic polyps.^33^ We found that an RDW >14.4% increased the locoregional recurrence rate 5.8 fold and was independently prognostic of DFS in patients with laryngeal cancer.

The reason for the association between a higher RDW and a poor outcome remains unclear. However, both inflammation and poor nutritional status may be in play.^24^, ^26^ RDW was positively associated with cancer stage in a study by Koma et al.^24^ We found a borderline significant interaction (*p* = 0.053); patients with advanced laryngeal cancer had significantly higher preoperative RDWs. However, we found no significant relationship between any other tumor characteristics associated with poor prognosis (extent of differentiation, nodal involvement, or perivascular and perineural invasion) in high-RDW patients.

It has been difficult to identify reliable predictors of locoregional recurrence after laryngeal cancer surgery. Brandstorp-Boesen et al.^34^ studied 1615 laryngeal cancer patients. Patients with supraglottic cancer, younger subjects, those with T2–T3 stage tumors, and patients treated earlier in the study period were at increased risks of recurrence. Haapaniemi et al.^35^ studied laryngeal cancer patients in Finland. Those with T2 glottic or T2 supraglottic cancer showed unexpectedly poor disease-specific survival. No clear explanation was apparent, but misclassifications among T2–T3 tumors and the lack of surgical intervention during management were suggested as explanations. We studied only patients who underwent surgery; Haapanemi et al.^35^ included patients undergoing either primary chemoradiotherapy or surgery. The high relapse frequency among patients with T2–T3 glottic carcinomas is in line with our results. Notably, total laryngectomy afforded the highest local control rate of (mostly) advanced tumors.

Our study has certain limitations. All work was performed at a single center, and our patient numbers were small. The work was retrospective in nature, and some data (particularly pathology reports) were lacking. Also, we did not explore whether RDW was associated with anemia, and we did not measure iron or vitamin B12 levels. However, RDW seems to be a significant predictor of laryngeal cancer prognosis. Thus, additional multicenter prospective studies with more patients are warranted.

## Conclusion

We found that the pretreatment RDW was independently prognostic of DFS in patients with laryngeal cancer. As the RDW is readily available, additional validation and feasibility studies are warranted to determine if the RDW is usually prognostic for laryngeal cancer. As the RDW is a routine (inexpensive) parameter, it may serve as a new, accurate, and reproducible means of identifying early-stage laryngeal cancer patients with poorer prognoses. When planning partial laryngectomy in the presence of RDW values higher than 14.4%, it has to be kept in mind that these patients are more likely to develop locoregional recurrences after surgery. However, additional prospective studies are needed.

## Conflicts of interest

The authors declare no conflicts of interest.

## References

[1] Forhecz Z., Gombos T., Borgulya G., Pozsonyi Z., Prohászka Z., Jánoskuti L. (2009). Red cell distribution width in heart failure: prediction of clinical events and relationship with markers of ineffective erythropoiesis, inflammation, renal function, and nutritional state. Am Heart J.

[2] Yesil A., Senates E., Bayoglu I.V., Erdem E.D., Demirtunç R., Kurdaş Övünç A.O. (2011). Red cell distribution width: a novel marker of activity in inflammatory bowel disease. Gut Liver.

[3] Lippi G., Targher G., Montagnana M., Salvagno G.L., Zoppini G., Guidi G.C. (2009). Relation between red blood cell distribution width and inflammatory biomarkers in a large cohort of unselected outpatients. Arch Pathol Lab Med.

[4] Jo Y.H., Kim K., Lee J.H., Kang C., Kim T., Park H.M. (2013). Red cell distribution width is a prognostic factor in severe sepsis and septic shock. Am J Emerg Med.

[5] Grant B.J.B., Kudalkar D.P., Muti P., McCann S.E., Trevisan M., Freudenheim J.L. (2003). Relation between lung function and RBC distribution width in a population-based study. Chest.

[6] Yazıcı P., Demir U., Bozdağ E., Bozkurt E., Işıl G., Bostancı Ö. (2015). What is the effect of treatment modality on red cell distribution width in patients with acute cholecystitis?. Ulusal Cer Derg.

[7] Mantovani A., Allavena P., Sica A., Balkwill F. (2008). Cancer-related inflammation. Nature.

[8] Balkwill F.R., Mantovani A. (2012). Cancer-related inflammation: common themes and therapeutic opportunities. Semin Cancer Biol.

[9] Hanahan D., Weinberg R.A. (2011). Hallmarks of cancer: the next generation. Cell.

[10] Kara M., Uysal S., Altinişik U., Cevizci S., Güçlü O., Dereköy F.S. (2017). The pre-treatment neutrophil-to-lymphocyte ratio, platelet-to-lymphocyte ratio, and red cell distribution width predict prognosis in patients with laryngeal carcinoma. Eur Arch Otorhinolaryngol.

[11] Bennett S.H., Futrell J.W., Roth J.A., Hoye R.C., Ketcham A.S. (1971). Prognostic significance of histologic host response in cancer of the larynx or hypopharynx. Cancer.

[12] Eskiizmir G., Tanyeri Toker G., Celik O., Gunhan K., Tan A., Ellidokuz H. (2017). Predictive and prognostic factors for patients with locoregionally advanced laryngeal carcinoma treated with surgical multimodality protocol. Eur Arch Otorhinolaryngol.

[13] You B., Gu M., Cao X., Li X., Shi S., Shan Y. (2017). Clinical significance of ADAM10 expression in laryngeal carcinoma. Oncol Lett.

[14] You Y., Yao H., You B., Li X., Ni H., Shi S. (2015). Clinical significance of HAX-1 expression in laryngeal carcinoma. Auris Nasus Larynx.

[15] Wu H., Xu H., Zhang S., Wang X., Zhu H., Zhang H. (2013). Potential therapeutic target and independent prognostic marker of TROP2 in laryngeal squamous cell carcinoma. Head Neck.

[16] Edge S.B., Byrd D.R., Compton C.C., Fritz A.G., Greene F., Trotti A. (2010).

[17] de Carvalho T.M., Miguel Marin D., da Silva C.A., de Souza A.L., Talamoni M., Lima C.S. (2015). Evaluation of patients with head and neck cancer performing standard treatment in relation to body composition, resting metabolic rate, and inflammatory cytokines. Head Neck.

[18] Campian J.L., Sarai G., Ye X., Marur S., Grossman S.A. (2014). Association between severe treatment-related lymphopenia and progression-free survival in patients with newly diagnosed squamous cell head and neck cancer. Head Neck.

[19] Rassouli A., Saliba J., Castano R., Hier M., Zeitouni A.G. (2015). Systemic inflammatory markers as independent prognosticators of head and neck squamous cell carcinoma. Head Neck.

[20] Kum R.O., Ozcan M., Baklacı D. (2014). Elevated neutrophil-tolymphocyte ratio in squamous cell carcinoma of larynx compared to benign and precancerous laryngeal lesions. Asian Pac J Cancer Prev.

[21] Zeng Y.-C., Chi F., Xing R., Xue M., Wu L.-N., Tang M.-Y. (2016). Pre-treatment neutrophil-to-lymphocyte ratio predicts prognosis in patients with locoregionally advanced laryngeal carcinoma treated with chemoradiotherapy. Jpn J Clin Oncol.

[22] Demirkol S., Balta S., Cakar M., Unlu M., Arslan Z., Kucuk U. (2013). Red cell distribution width: a novel inflammatory marker in clinical practice. Cardiol J.

[23] Periša V., Zibar L., Sinčić-Petričević J., Knezović A., Periša I., Barbić J. (2015). Red blood cell distribution width as a simple negative prognostic factor in patients with diffuse large B-cell lymphoma: a retrospective study. Croat Med J.

[24] Koma Y., Onishi A., Matsuoka H., Oda N., Yokota N., Matsumoto Y. (2013). Increased red blood cell distribution width associates with cancer stage and prognosis in patients with lung cancer. PLoS ONE.

[25] Howell-Burke D., Peters L.J., Goepfert H., Oswald M.J. (1990). T2 glottic cancer: recurrence, salvage, and survival after definitive radiotherapy. Arch Otolaryngol Head Neck Surg.

[26] Ward M.C., Adelstein D.J., Bhateja P., Nwizu T.I., Scharpf J., Houston N. (2016). Severe late dysphagia and cause of death after concurrent chemoradiation for larynx cancer in patients eligible for RTOG 91-11. Oral Oncol.

[27] Lippi G., Salvagno G.L., Guidi G.C. (2014). Red blood cell distribution width is significantly associated with aging and gender. Clin Chem Lab Med.

[28] Alis R., Fuster O., Rivera L., Romagnoli M., Vaya A. (2015). Influence of age and gender on red blood cell distribution width. Clin Chem Lab Med.

[29] Lippi G., Sanchis-Gomar F., Montagnana M. (2014). Biological markers in older people at risk of mobility limitations. Curr Pharm Des.

[30] Salvagno G.L., Sanchis-Gomar F., Picanza A., Lippi G. (2015). Red blood cell distribution width: a simple parameter with multiple clinical applications. Crit Rev Clin Lab Sci.

[31] Yazıcı P., Demir U., Bozkurt E., Işıl G., Mihmanlı M. (2017). The role of red cell distribution width in the prognosis of patients with gastric cancer. Cancer Biomark.

[32] Riedl J., Posch F., Konigsbrugge O., Lötsch F., Reitter E.M., Eigenbauer E. (2014). Red cell distribution width and other red blood cell parameters in patients with cancer: association with risk of venous thromboembolism and mortality. PLOS ONE.

[33] Ay S., Eryilmaz M.A., Aksoy N., Okus A., Unlu Y., Sevinc B. (2015). Is early detection of colon cancer possible with red blood cell distribution width?. Asian Pac J Cancer Prev.

[34] Brandstorp-Boesen J., Sørum Falk R., Folkvard Evensen J., Boysen M., Brøndbo K. (2016). Risk of recurrence in laryngeal cancer. PLOS ONE.

[35] Haapaniemi A., Koivunen P., Saarilahti K., Kinnunen I., Laranne J., Aaltonen L.M. (2016). Laryngeal cancer in Finland: a five-year follow-up study of 366 patients. Head Neck.

